# Psychometric Properties of Geriatric Depression Scale (Malay Version) in Elderly with Cognitive Impairment

**DOI:** 10.21315/mjms2021.28.3.9

**Published:** 2021-06-30

**Authors:** Azlina Wati Nikmat, Zahir Izuan Azhar, Norley Shuib, Nurul Azreen Hashim

**Affiliations:** 1 Department of Psychiatry, Faculty of Medicine, Universiti Teknologi MARA, Sungai Buloh, Selangor, Malaysia; 2 Department of Public Health Medicine, Faculty of Medicine, Universiti Teknologi MARA, Sungai Buloh, Selangor, Malaysia

**Keywords:** depressive symptoms, elderly, cognitive dysfunction, validation, cognitive decline

## Abstract

**Background:**

Geriatric Depression Scale (GDS) has been widely used in assessing depression in elderly population. Using the Ministry of Health Malaysia definition for elderly, this study aimed to examine the construct validity and reliability of the GDS (Malay version) in elderly with cognitive impairment in Malaysia.

**Methods:**

A cross-sectional validation study involving 219 elderlies with cognitive impairment was conducted using universal sampling method. Participants aged 60 years old and above, scored less than 11 for the short mini mental state examination (SMMSE), have sufficient command of the Malay or English language, residing in the government nursing homes and attending memory clinics in government hospitals in Klang Valley were sampled. The GDS-15 English version underwent adaptation, translation, face validation and field testing to produce the Malay version. Psychometric analysis was performed using the exploratory factor analysis and its internal consistency was examined.

**Results:**

Mean age of participants were 72.61 ± 7.79. Majority were male (50.7%), Malay (50.2%), studied at least until primary school (71.7%) and stayed at nursing homes (50.2%). Construct validity showed significant Bartlett’s test of sphericity (Chi-square = 1,340.058, *P* < 0.001) and Kaiser-Meyer-Olkin (KMO) test of 0.90. Factor loadings for each item in the depression domain were satisfactory ranging from 0.42 to 0.83. Factor loadings for each item in the psychosocial activities’ domain were satisfactory ranging from 0.53 to 0.76. For the questionnaire reliability analysis, the total Cronbach’s alpha for the final model was satisfactory, with and overall Cronbach’s alpha of 0.89. Cronbach’s alpha value for the depression and psychosocial activities domain was 0.861 and 0.80, respectively.

**Conclusion:**

The GDS (Malay version) is a valid and reliable tool to screen for depression in elderly with cognitive impairment.

## Introduction

Depression in elderly is a serious mental illness with significant morbidity and mortality. Prevalence of depression in elderly ranges between 1% and 49%, depends on the sample population and the scales used in the studies (1). In Malaysia, prevalence of depression in elderly was found to be around 7.6% (2) to 30% (3). Bereavement, sleep disturbance, disability, previous history of depression (4), somatic illness, cognitive and functional impairment, lack of close social contacts and female gender were found to be risk factors of depression in elderly (1). Depression in this population will worsen the pre-existing medical conditions, decrease physical, cognitive and social functioning, cause greater self-neglect and increase risk of suicide (5).

Depression in elderly is still underdiagnosed and undertreated due to misattribution of symptoms to normal aging, misdiagnosis and social stigma (6). There are different types of scales used to screen for depression in elderly population, for example, Geriatric Depression Scale (GDS) (7), Center for Epidemiologic Studies-Depression Scale (CES-D) (8) and Cornell Scale for Depression in Dementia (5).

The GDS is a self-rating scale developed to screen for depression in older adult populations. Items in GDS seek information representing lowered affect, decreased activity levels, irritability, withdrawal, distressing thoughts, and negative judgments about the past, present and future. It is presented in a yes/no response format with one point assigned to each yes answer. Items are summed and higher scores indicate higher depression level (7). The original version of the GDS consists of 30 items (GDS-30) while short versions of the GDS contains 15 items (GDS-15), 10 items (GDS-10) or 4 items (GDS-4) (9).

GDS-15 is most widely used in studies and clinical practice due to its practicality and it performs as well as the longer version (10). Total scores for the GDS-15 ranged from 0 to 15, respectively. For GDS-15, scores of 0–4 are considered normal (depending on age, education and complaints), 5–9 indicates mild depression and 10–10 indicates severe depression (11). Internal consistency of GDS-15 was reported around 0.80 (11). The use of the cut-off point 4/5 for the GDS-15 produced sensitivity and specificity rates of 92.7% and 65.2%, respectively, when ICD-10 diagnostic criteria for major depressive episode were used as the ‘gold standard’ (9).

Both versions of the GDS (GDS-30, GDS-15) have been translated and validated across different clinical settings, cultures and countries including Malaysia (12–15). In the Malaysian population, a validation study done by Teh and Hasanah (16) omitted item-9 in GDS-15 to form the new Malay GDS-14 scale (M-GDS-14). M-GDS-14 revealed good psychometric purposes with Cronbach’s alpha coefficient of 0.84 and test-retest reliability of 0.84 (15). Though the GDS-15 has been translated and validated in Malaysian population, most of the study were done on general elderly population. In this study, we would like to examine the validity and reliability of the scale on elderly with cognitive impairment.

Hence, the objective of the current study is to translate and validate the GDS-15 and to produce a well-adapted Malay version of GDS-15 for elderly with cognitive impairment and to evaluate the psychometric properties of GDS-15.

## Methods

### Participants and Procedure

This study involves elderly with cognitive impairment, which were recruited from government nursing homes and memory clinics in government hospitals. Elderly in this study was defined using the United Nations and the Ministry of Health Malaysia’s recommendation as those with a chronological age of 60 years old and above (17, 18). The two nursing homes that participated in the study were the Rumah Seri Kenangan Cheras and the Rumah Ehsan. As for the government hospitals, participants were recruited from Hospital Selayang and Hospital Sungai Buloh, Selangor.

Managers of the nursing homes provided list of residents in their respective settings. All residents were approached and invited to participate in the study. As for government hospitals, the nurses from the memory clinics provided list of patients who attended the memory clinic from June until October 2015. Consenting participants from both settings were then assessed using the short mini mental state examination (SMMSE). Responses from the SMMSE were scored at that particular time. This assessment was administered by the researcher herself, who is a registered clinical psychologist. Participants who scored below 11 and able to communicate or understand Malay or English languages were recruited in the study. A total of 269 participants meet the study criteria and randomly assigned to Phase 2 and Phase 3 groups.

### Sample Size

The sample size calculation was based on sample to variable ratio (SVR), denoted as *N*:*p* ratio, where *N* refers to the number of participants and *p* refers to the number of items studied. *N*:*p* ratio recommendation ranges from 3:1 to 20:1 (19). The GDS questionnaire contains 15 items and this study used the *N*:*p* ratio of 10:1. Therefore, the minimum sample size required is 150 participants. Taking into account the attrition rate of 10%, data collection required a minimum of 165 participants. However, the final total number of samples was more than the minimum requirement, which was 269 participants.

## Measurements

### Demographic Data

Socio-demographic questions included age, gender, ethnicity, marital status, education attained and financial status.

### SMMSE

The SMMSE (1) is a brief cognitive screening tool derived from the original Mini Mental State Examination (MMSE) (2). It consists of 12 items and is scored binomially which gives a total score out of 12. The cut off score of 10 was suggested by the original author to differentiate those with cognitive impairment from the normal population with a reported sensitivity of 98% and specificity of 91% (1).

### GDS-15

GDS-15 is a self-rating scale developed to screen depression in elderly’ population (3). The original version of GDS consists of 30 items (GDS-30), while a short version of the GDS contains 15 items (GDS-15). These items seek information representing lowered affect, decreased activity levels, irritability, withdrawal, distressing thoughts, and negative judgments about the past, present and future (4). It is presented in a yes/no response format with one point is assigned to each answer. Items were summed and higher scores indicate higher depression level. Total scores for GDS-15 ranged from 0 to 15, respectively (20).

## Study Design and Procedure

This was a cross-sectional validation study conducted in three phases: i) Phase 1: Adaptation and translation of the GDS from the original English language into the Malay language; ii) Phase 2: Face validation of the GDS (Malay version) and iii) Phase 3: Field testing and psychometric analysis of the GDS (Malay version).

- Phase 1: Adaptation of the GDS (Malay version) from the original English version was done by the Institute of Translation of Malaysia in accordance to the WHO guideline of translating and adapting of instruments in which, two independent native Malay language experts carried out the forward translation whose quality was checked by another set of independent translators. The backward translation into English language was carried out by another two independent translators. Discrepancies out of this process were resolved and consensus reached about the harmonised Malay version of GDS-15. The final version of translated questionnaire was approved by the Institute of Translation of Malaysia and researchers were consulted by the panel during this process.
- Phase 2: The GDS-15 (Malay version) underwent face validation on the target population. Sample size of 30 or more are recommended for face validation (21). For this study, 50 elderlies with cognitive impairment that has been identified during selection process above was randomly selected using online random number generator (1–269). Participants were administered on the GDS (Malay version). However, one participant was removed during analysis due to missing values during data collection. Therefore, the final total sample for this part was 49 participants.
  The face validation was conducted to assess their understanding of the purpose, content, wording, instructions and general structure of the GDS-15 (Malay version). Correction and fine tuning of the GDS-15 (Malay version) by the research team was done based on the participants’ feedback. The feedback obtained showed that the questionnaire was satisfactory and no further amendment was required. The face validated GDS-15 (Malay version) was ready for field testing. Participants who took part in Phase 2 (face validation) and Phase 3 (field testing) were mutually exclusive, as those who participated for the Phase 2 were not re-selected for the Phase 3 of this study.
- Phase 3, the GDS-15 (Malay version) was field tested on the remaining 219 elderlies with cognitive impairment, which has been identified during selection process above. Participants were then provided with a socio demographic and GDS-15 (Malay version) questionnaires (19).

## Statistical Analysis

Data were analysed using the Statistical Package for the Social Sciences version 24.0. Missing values and data entry errors were checked prior to analysis. Descriptive statistics were presented as frequency and percentage for categorical data and mean with standard deviation for continuous data. Normality of the data was assessed through frequencies and data distribution of the GDS scales.

Exploratory factor analysis was used to investigate the factor structure of the GDS-15 using a factor extraction criterion of eigenvalues greater than 1.00. Principal axis factoring with varimax rotation was used with the assumption that the resultant factors are correlated. The pattern matrix was then examined for the loadings and cross loading, with an item loading of more than 0.4 was considered significant to be retained providing that it does not load highly to other factors. The adequacy for factor analysis was checked using the Kaiser-Meyer-Olkin (KMO) and Bartlett’s tests. Value closer to 1 for the KMO test indicates that factors resulted from the factor analysis is distinct and reliable (22). The level of significance for the Bartlett’s test was set at *P* < 0.05.

Reliability of the GDS-15 was assessed by examining the internal consistency of the scales using Cronbach’s alpha coefficient. The reliability was considered to be good if the alpha (α) value was above 0.7 (11).

## Results

There were 219 elderly Malaysians who participated in this research. The mean age of respondents is 72.61 ± 7.79. Majority were male (50.7%), Malay (50.2%), studied at least until primary school (71.7%) and stayed at nursing homes (50.2%) (Table 1).

Construct validity results through exploratory factor analysis showed significant Bartlett’s test of sphericity (Chi-square = 1,340.058, *P* < 0.001) and KMO test of 0.903. The Kaiser’s criterion showed two factors with Eigenvalues of ≥ 1 (values of 6.197 and 1.495), with the total variance of 51.28%. Using the principal component analysis with Varimax rotation, two factor domains were identified.

Ten items were loaded into the first domain while five items were loaded into the second domain. The first domain is the depression domain that consists of 10 questions. Factor loadings for this domain for each item were satisfactory ranging from 0.421 to 0.833. The second domain is the psychosocial activities domain that consists of five questions. Factor loadings for this domain for each item were satisfactory ranging from 0.539 to 0.768. Therefore, this model was considered as the final model (Table 2) and none of the items were removed or added to get the final model.

For the questionnaire reliability analysis, the total Cronbach’s alpha for the final model total was satisfactory. The overall value for Cronbach’s alpha was 0.890. Cronbach’s alpha value for the first and second domain are 0.861 and 0.806, respectively (Table 3).

## Discussion

This study validated the GDS-15 (Malay version) which involved 219 elderlies with cognitive impairment (23). It was conducted in a different population compared to the previous one done which involved elderly inpatients of a teaching hospital in Malaysia, which excluded those with significant cognitive impairment (MMSE score < 24) (24). In contrast, our participants were recruited from government nursing homes and community home care residents and included those with cognitive impairment.

The final validated GDS-15 (Malay version) consisted of two domains which are depression and psychosocial activities. The depression domain had ten items and the psychosocial domain had five items making the total 15 items. Factor loadings for each item in both domains were found to be satisfactory. This differs from the previous validation study of the GDS (Malay version) where they omitted the item “Do you prefer to stay at home, rather than going out and doing new thing?” The item was found to be poorly correlated with corrected item total score and had no discriminatory value against clinical diagnosis of major depression and all clinically significant depression. A new scale was suggested with only 14 items (24).

Cronbach’s alpha value for the depression domain was 0.861 and psychosocial activities domain was 0.806. The overall value of 0.890 which indicated of a high degree of internal consistency. This is consistent with other studies done in Asian countries, which reported a Cronbach alpha ranging from 0.81–0.84 (24–26).

There is limitation in this validation study. The diagnosis of depression in participants was not confirmed using gold standard criteria for example the DSM-IV-TR criteria for depression. Additionally, test retest reliability was not performed. However, the study has a large sample size and consisted of elderly respondents from different settings (those who were attending memory clinic either living with a carer or in nursing homes).

## Conclusion

In conclusion, elderly people with cognitive impairment need to be routinely screened for depression using a short, feasible and conveniently administered tool regardless of the clinical setting. The GDS-15 (Malay version) is a reliable and valid tool for this purpose.

## Figures and Tables

**Table 1:** Respondents sociodemographic profile

Variable (*n* = 219)	*n* (%)	Mean (±SD)
Gender		
Male	111 (50.7)	
Female	108 (49.3)	
Age		72.61 ± 7.79
Race		
Malay	110 (50.2)	
Chinese	63 (28.8)	
Indian	44 (20.1)	
Others	2 (0.9)	
Education		
No formal education	62 (28.3)	
Primary school	134 (61.2)	
Secondary school	23 (10.5)	
Living arrangements		
Living at home	109 (49.8)	
Nursing home	110 (50.2)	

Note: *n* = frequency; SD = standard deviation

**Table 2:** Exploratory factor analysis of GDS (final model)

Items	1	2
***Domain 1: Kemurungan***		
*Adakah anda pada asasnya berpuas hati dengan kehidupan anda?*	0.727	
*Adakah anda berasa hidup anda kekosongan?*	0.546	
*Adakah anda berasa gembira dalam kebanyakan masa?*	0.608	
*Adakah anda bimbang sesuatu yang buruk akan terjadi pada anda?*	0.421	
*Adakah anda berasa gembira dalam kebanyakan masa?*	0.585	
*Adakah anda berasa bahawa anda mempunyai lebih banyak masalah daya ingatan daripada orang lain?*	0.475	
*Adakah anda fikir alangkah baiknya untuk hidup sekarang?*	0.833	
*Adakah anda berasa keadaan anda sekarang kurang berguna?*	0.730	
*Adakah anda berasa keadaan anda tidak ada harapan?*	0.667	
*Adakah anda fikir bahawa kebanyakan orang adalah lebih baik daripada anda?*	0.660	
***Domain 2: Aktiviti psikososial***		
*Adakah anda telah meninggalkan banyak kegiatan dan minat anda?*		0.768
*Adakah anda sering berasa tidak terdaya?*		0.722
*Adakah anda sering berasa bosan?*		0.685
*Adakah anda lebih suka duduk di rumah daripada keluar dan melakukan sesuatu perkara/hal yang baru?*		0.747
*Adakah anda berasa penuh bertenaga?*		0.539

Note: Bartlet’s test of sphericity (Chi-square = 1,340.058, *P* < 0.001) and KMO test = 0.903

**Table 3:** Reliability analysis of Cornell scale for depression in dementia (final model)

Domain	Cronbach’s alpha
Domain 1: (10 items)	
Depression	0.861
Domain 2: (5 items)	
Psychosocial activities	0.806

Total	0.890
